# Phylogeography of *Prunus armeniaca* L. revealed by chloroplast DNA and nuclear ribosomal sequences

**DOI:** 10.1038/s41598-021-93050-w

**Published:** 2021-07-01

**Authors:** Wen-Wen Li, Li-Qiang Liu, Qiu-Ping Zhang, Wei-Quan Zhou, Guo-Quan Fan, Kang Liao

**Affiliations:** 1 College of Horticulture and Forestry, Xinjiang Agricultural University, Urumqi, Xinjiang China; 2 Xiongyue National Germplasm Resources Garden of the Liaoning Institute of Pomology, Xiongyue, Shenyang, China; 3 Luntai National Fruit Germplasm Resources Garden of Xinjiang Academy of Agricultural Sciences, Luntai, Xinjiang China

**Keywords:** Ecology, Evolution, Genetics, Plant sciences

## Abstract

To clarify the phytogeography of *Prunus armeniaca* L., two chloroplast DNA fragments (*trn*L-*trn*F and *ycf*1) and the nuclear ribosomal DNA internal transcribed spacer (ITS) were employed to assess genetic variation across 12 *P. armeniaca* populations. The results of cpDNA and ITS sequence data analysis showed a high the level of genetic diversity (cpDNA: *H*_T_ = 0.499; ITS: *H*_T_ = 0.876) and a low level of genetic differentiation (cpDNA: *F*_*ST*_ = 0.1628; ITS: *F*_*ST*_ = 0.0297) in *P. armeniaca*. Analysis of molecular variance (AMOVA) revealed that most of the genetic variation in *P. armeniaca* occurred among individuals within populations. The value of interpopulation differentiation (*N*_*ST*_) was significantly higher than the number of substitution types (*G*_*ST*_), indicating genealogical structure in *P. armeniaca*. *P. armeniaca* shared genotypes with related species and may be associated with them through continuous and extensive gene flow. The haplotypes/genotypes of cultivated apricot populations in Xinjiang, North China, and foreign apricot populations were mixed with large numbers of haplotypes/genotypes of wild apricot populations from the Ili River Valley. The wild apricot populations in the Ili River Valley contained the ancestral haplotypes/genotypes with the highest genetic diversity and were located in an area considered a potential glacial refugium for *P. armeniaca*. Since population expansion occurred 16.53 kyr ago, the area has provided a suitable climate for the population and protected the genetic diversity of *P. armeniaca*.

## Introduction

The evolutionary history of organisms, including their genetic diversity, population structure and historical dynamics, is critical to species conservation^1^. Understanding the effects of climate change on the spatial genetic patterns of species, particularly endangered species, can help reveal not only the evolutionary history of species but also conservation strategies^2,3^. The origins of mountain biodiversity are complex and may include the immigration of preadapted lineages^4–5^, in situ diversification^6^, or the continuation of ancestral lineages^7^. Compared with those of other mountains, such as the Hengduan Mountains, the organismal evolution and diversity of the Tianshan Mountains are still poorly understood. The Ili River Valley is located in the western part of the Tianshan Mountains in China and is surrounded by mountains on three sides. This valley was the main northern crossing of the ancient Silk Road. In the late Tertiary period, a large number of species, including wild apricot, wild apple and wild hawthorn, remained in the Ili River Valley and were important components of the deciduous broad-leaved forest at elevations below the coniferous forest and above the mountain grassland belt in the Xinjiang Uygur Autonomous Region, China^8^. However, there have been few studies on the phylogeography of plant species in the arid region of Northwest China^9–10^.

In recent decades, the method of combining molecular data with paleoclimatic and geographical evidence has been effective in the study of systematic geography^11–12^. Many systematic geographic studies have shown that the Last Glacial Maximum (LGM) of the Pleistocene strongly influenced the genetic variation and biodiversity of plants throughout the Northern Hemisphere^13–14^. Especially during the Quaternary glacial period, species in the ice-free zone were mainly affected by the cold and dry climate^15^. Climatic fluctuations cause the distribution of species to shrink and expand^16^, and cold and dry climates prompt plant and animal species to retreat to refugia, which provided shelter^17^. As temperatures rose after the glacial period, species underwent population expansion, changes that left a genetic signature in their current populations^18^. Although no unified glacial sheet was present in Asia, paleodata suggest that the distribution ranges of woody species in this region were similarly changed by Quaternary climatic oscillations^19^. Based on the geographical distribution of genetic variation, the glacial refuges and postglacial revegetation routes of most woody plants are roughly consistent with fossil evidence^20^. However, due to the lack of fossil evidence, genetic evidence has become an important means of providing information on the distribution range of some woody species and the history of glacial refugia^21–22^. Volkova^23^ interpreted the observed genetic structure [nuclear (internal transcribed spacer, ITS) and plastid DNA] of the Eurasian populations of *Prunus padus* as plausibly resulting from at least two cycles of glacial survival in refugia followed by postglacial colonization events^17^. The complex geographic history in the area may have provided a refuge for species in the last glacial period^17^. Xu et al.^24^ proposed two possible independent glacial refugia in Northwest China: the Ili River Valley and the Northern Junggar Basin. Su et al.^9^ speculated that intensification of the dry and cold climate during the early Quaternary, combined with plant physiological features, contributed to the lineage split, and climate oscillations most likely led to the Ili range expansion. Therefore, it is of great interest to study the systematic geography of endemic plants in Northwest China against the background of Quaternary climatic fluctuations.

Apricot is a fruit of temperate and subtropical regions. Turkey, Uzbekistan, Iran, Algeria, Italy, Spain, China, Pakistan, France and Japan are the main producers of apricots (http://www.fao.org/home/zh). Apricot belongs to section *Armeniaca* (Lam.) Koch, subgenus *Prunophora* Focke and genus *Prunus* (Rosaceae)^25^. Almost all cultivated apricots originated from *Prunus armeniaca*^26^. In China, wild apricot is distributed in the Ili River Valley (Tianshan Mountain area). The species is also distributed along the Tianshan Mountains and westward to Kazakhstan, Kyrgyzstan and Uzbekistan. It is a relic of a broad-leaved forest from the late Tertiary period, which played a decisive role in the domestication of cultivated apricots worldwide^27^. As the world experienced extreme weather events, especially glacial periods, and most species went extinct, some of the more complex valleys may have become refugia for surviving forests and local species. As a result, traces of the species may be gradually shrinking in such areas. Therefore, is the Ili River Valley a glacial refugium for wild apricot?

Generally, the dispersal distance of seeds is much shorter than that of pollen, and population divergence due to genetic drift will be more marked for chloroplast DNA (cpDNA) than for nuclear DNA. Indeed, cpDNA is considered to evolve very slowly, with low recombination and mutation rates^28^. Organelle markers could provide powerful tools for studying the phylogeography and migratory footprints of species^29^. Parental genetic markers are often combined with single-parent organelle markers for population genetics studies^30^. cpDNA and ITS sequence variations have been very effective in revealing the glacial refuges of plants^29^. cpDNA lineages usually show the unique geographical distribution and evolutionary history of natural populations and are therefore widely used in systematic geography studies^31^. Li et al.^32^ successfully used cpDNA and ITS markers to evaluate the diversity and phylogenetic relationships of populations of *Saxifraga sinomontana*, indicating that the species had microrefugia during the Quaternary glacial period. In this study, we employed cpDNA (*trn*L*-trn*F and *ycf*1) and nuclear ribosomal DNA (nrDNA) sequences to (1) reveal the haplotype/genotypic diversity and population genetic structure of the species and (2) examine the demographic history of *P. armeniaca* during Quaternary climate oscillations, and further explore the origin and evolution of this species*.*

## Materials and methods

### Sample collection

The samples used for cpDNA analysis included 123 individuals from 20 populations. A total of 171 individuals from 19 populations were used for the ITS analysis, of which 38 samples were obtained from the NCBI database (Table S1). The samples studied were from *P. armeniaca* and related species (*Prunus sibirica,* NAG; *Prunus mandshurica*, LX; *Prunus dasycarpa*, ZX; *Prunus mume*, ECG; *Prunus zhengheensis*, ZHX^33^; *Prunus limeixing*, LMX^33^ and *Prunus brigantina*, FGX). *Prunus davidiana* (T) was used as the outgroup.

Our collection of wild apricot (*P. armeniaca*) populations covered most of the natural distribution in China, including Huocheng County (DZGhcmd, DZGhcy and DZGhcm populations), Yining County (DZGyn population), Gongliu County (DZGglb and DZGgld populations) and Xinyuan County (DZGxyt, DZGxya and DZGxyz populations). The distance between individuals sampled in each population was at least 100 m. Young leaves were collected and dried immediately with silica gel.

The cultivated populations of *P. armeniaca* included the Xinjiang apricot group (CAG, cultivated apricots in Xinjiang), the North China apricot group (NCG, cultivated apricots in Shandong, Shaanxi, Gansu, Liaoning and Ningxia) and the foreign apricot group (EG, cultivated apricots in the USA, France, Italy and Australia). Detailed sample information is provided in Table 1 and Table S2. The main characteristics of different populations can be found in Zhang et al.^8^.

**Table 1:** Population code, sampling location, coordinates, altitude and types of *P. armeniaca* and relative species.

Population	Origin/location	Type	Latitude (N°)	Longitude (E°)	Altitude (m)
***P. armeniaca***					
DZGhcmd	Huocheng, Xinjiang	W	44.43	80.79	1187.6
DZGhcy	Huocheng, Xinjiang	W	44.44	80.79	1245.7
DZGhcm	Huocheng, Xinjiang	W	44.40	80.71	1244.1
DZGyn	Yining, Xinjiang	W	44.12	81.62	1983.6
DZGglb	Gongliu, Xinjiang	W	43.25	82.86	1371.5
DZGgld	Gongliu, Xinjiang	W	43.23	82.75	1269.4
DZGxyt	Xinyuan, Xinjiang	W	43.54	83.44	1138.8
DZGxya	Xinyuan, Xinjiang	W	43.50	83.70	1275.3
DZGxyz	Xinyuan, Xinjiang	W	43.38	83.61	1374.1
CAG	Luntai, Xinjiang	C	41.78	84.23	972.0
NCG	Luntai, Xinjiang	C	41.78	84.23	972.0
EG	Xiongyue, Liaoning	C	40.17	122.16	20.4
***P. sibirica***					
NAG	Xiongyue, Liaoning	W	40.17	122.16	20.4
***P. mandshurica***					
LX	Xiongyue, Liaoning	C	40.17	122.16	20.4
***P. dasycarpa***					
ZX	Luntai, Xinjiang	C	41.78	84.23	972.0
***P. mume***					
ECG	Xiongyue,Liaoning	C	40.17	122.16	20.4
***P. zhengheensis***					
ZHX	Xiongyue, Liaoning	C	40.17	122.16	20.4
***P. limeixing***					
LMX	Xiongyue, Liaoning	C	40.17	122.16	20.4
***P. brigantina***					
FGX	Xiongyue, Liaoning	C	40.17	122.16	20.4
***P. davidiana***					
T	Luntai, Xinjiang	O	41.78	84.23	972.0

W, wild; C, cultivars; O, out group.

### DNA sequencing

Total genomic DNA was extracted from the silica gel-dried leaf materials using a Plant Genomic DNA Kit (Tiangen Biotech, Beijing, China)^34^. The quality and concentration of the extracted DNA were determined by 1% agarose gel electrophoresis and ultraviolet spectrophotometry, respectively.

cpDNA and nrDNA sequences from 15 samples were initially screened using universal primers. The sequencing results showed that the sequences of cpDNA (genes *trn*L*-trn*F and *ycf*1) and two nuclear ribosomal ITS regions (ITS1 and ITS2) were polymorphic. cpDNA and ITS fragments were amplified by polymerase chain reaction (PCR), and the details of their primers are provided in Table S3^35–37^. PCR was performed in a total volume of 25 µL that contained 1 µL DNA, 5.5 µL PCR mix, 16.5 µL double-distilled water and 1 µL each forward or reverse primer. PCR amplifications were performed under the following conditions: 5 min of initial denaturation at 94 °C and 35 cycles of 0.5 min at 94 °C, 0.5 min of annealing at 58°, and 0.5 min of extension at 72 °C, with 10 min of final extension at 72 °C. A CASpure PCR Purification Kit (CASarray, Shanghai, China)^32^ was used for purification. The purified PCR products were sequenced on an ABI PRISM 3730XL DNA Analyzer (Applied Biosystems, Foster City, CA, USA)^38^.

### Data analysis

We used BioEdit^39^ to view and manually correct the sequencing results. We first used CLUSTAL W^40^ to align the sequences and coded indels following the method of Simmons and Ochoterena^41^. Then, manual adjustments were made in MEGA ver. 7.0.26^42^ to remove the overhanging tails and ensure a uniform sequence length. We concatenated two chloroplast fragments (*trn*L-*trn*F and *ycf*1) into a separate matrix for subsequent analysis. We used DnaSP ver. 5.10 to identify different haplotypes (cpDNA sequences) or genotypes (ITS sequences). A haplotype network was constructed using TCS ver. 1.2.1^43^. ArcGIS ver. 10.2 (http://desktop.arcgis.com) software was used to create a haplotype geographical distribution map.

Haplotype/genotype diversity (*Hd*) and nucleotide diversity (π) were calculated using DnaSP ver. 5.10 software^44^. The within- population gene diversity (*H*_*S*_), gene diversity in all populations (*H*_*T*_), interpopulation differentiation (*G*_*ST*_) and number of substitution types (*N*_*ST*_) were calculated using PERMUT^45^ ver. 2.0. The last two indexes (*G*_*ST*_ and *N*_*ST*_) were analyzed via permutation tests with 1000 permutations. When *N*_*ST*_ is greater than *G*_*ST*_, it indicates the existence of genealogical geographic structure^45^. Analysis of molecular variance (AMOVA) was performed using Arlequin ver. 3.5.2.2^46^ to partition the genetic variation at different levels, with statistical significance determined by 1,000 permutations.

To investigate the historical dynamics of *P. armeniaca*, mismatch distribution analysis was conducted using DnaSP ver. 5.10. The sum of squared deviations (SSDs), Harpending's raggedness index (HRI)^45^ and corresponding *P* values were calculated using Arlequin ver. 3.5.2.2^46^. Neutrality tests based on Tajima’s *D* and Fu’s *F*_*S*_ were conducted to detect departures from the population equilibrium by Arlequin ver. 3.5.2.2^46^. According to the formula of Rogers and Harpending^47^, T = τ/2u, where “τ” is the parameter value from the mismatch distribution model. In the formula u = μkg, “μ” is the base substitution rate (chloroplast angiosperms^48^: 1.1 × 10^–9^), “k” is the fragment length (cpDNA length after combination: 2062 bp), and “g” is the generation time (20 years)^49^.

The outgroup was *P. davidiana*, and the time point of peach-apricot differentiation was used as the calibration point^50^. The BEAUti interface was used to create an input file for BEAST^51^, for which the GTR + I + G nucleotide substitution model was used. The data were analyzed using a relaxed log-normal clock model and the Yule process speciation model for the tree priors. Prior settings for calibrating nodes were an offset of 55.1 Ma and a log mean of 1.0 (log stdev of 0.5). The Bayesian Markov chain Monte Carlo simulation was run for 100 million generations with a sample frequency of 1000, and the first 20% of generations were discarded as burn-in. Three independent analyses were conducted and their results combined by LogCombiner ver. 1.8.4. Finally, annotation and visualization of the maximum clade credibility tree were performed in TreeAnnotator 1.8.4 and FigTree ver. 1.4.3, respectively.

## Results

### Haplotype/genotype phylogenetics and distribution

**Figure 1:**
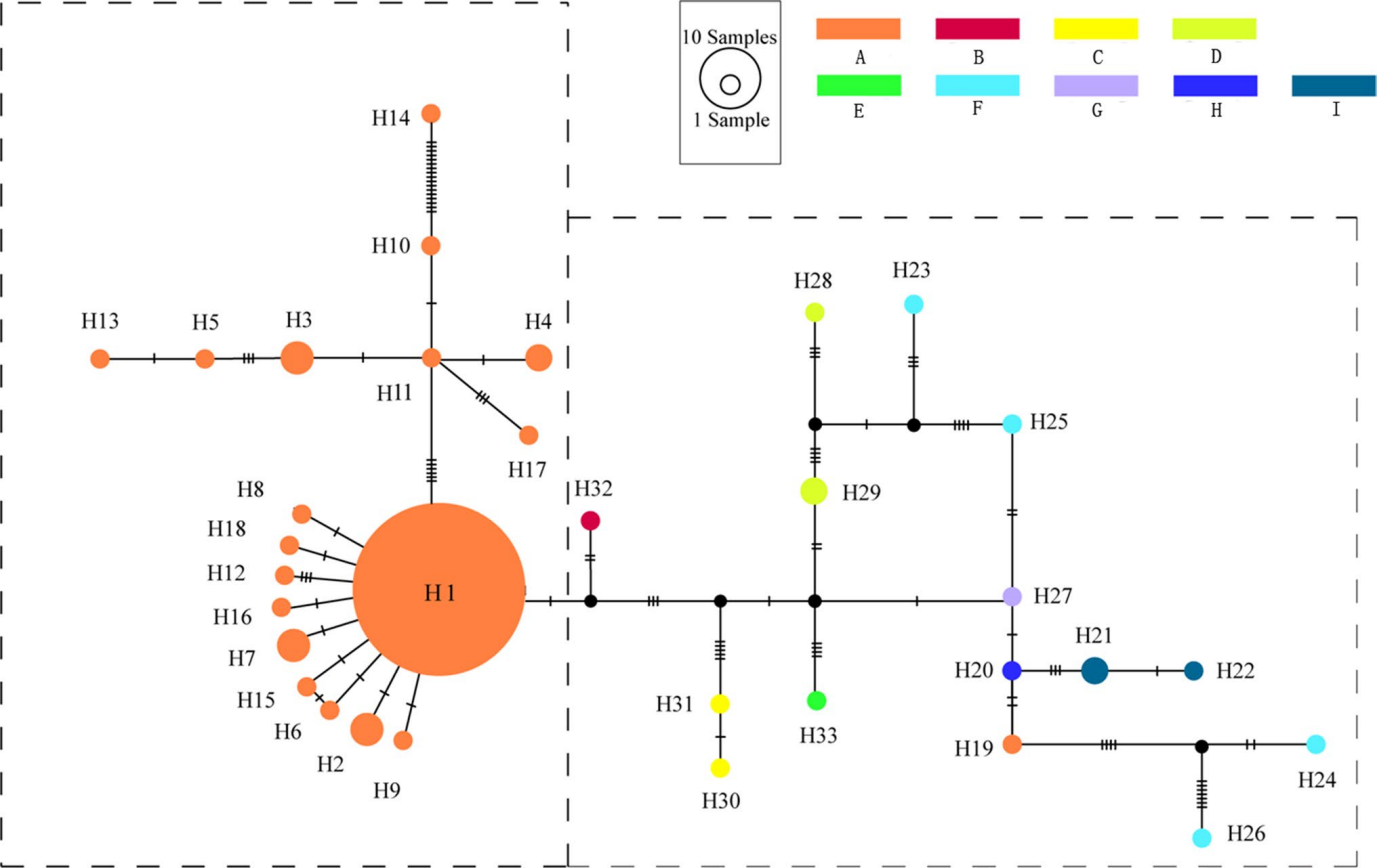
The haplotype network generated from the haplotypes of *P. armeniaca* and related species based on cpDNA dataset. The small black circles shown an intermediate haplotype not detected in this study. (**A**) *P. armeniaca*; (**B**) *P. zhengheensis*; (**C**) *P. davidiana*; (**D**) *P. sibirica*; (**E**) *P. dasycarpa*; (**F**) *P. limeixing*; (**G**) *P. mandshurica*; (**H**) *P. mume*; (**I**) *P. brigantina.* The haplotype network was constructed using TCS ver. 1.2.1 and then improved in Adobe Illustrator CC (Adobe Systems Inc., CA, USA).

Based on the concatenated cpDNA sequences (*trn*L*-trn*F and *ycf*1), 33 haplotypes (H1-H33) were identified among 123 individuals from 20 populations of *P. armeniaca* and related species (Fig. 1, Table 2). The alignment lengths of the two chloroplast fragments were 746 bp and 1316 bp, respectively, and the combined length was 2026 bp. Variable sites among the 33 haplotypes are shown in Table S4. *P. armeniaca* harbored haplotypes H1-H17 and H19-H20; *P. sibirica* harbored haplotypes H28-H29; *P. mandshurica* harbored haplotypes H27; *P. dasycarpa* harbored haplotypes H33; *P. mume* harbored haplotypes H20; *P. zhengheensis* harbored haplotypes H32; *P. limeixing* harbored haplotypes H23–H26; *P. brigantina* harbored haplotypes H21–H22; and *P. davidiana* harbored haplotypes H30–H31. The results showed that populations of different species did not share haplotypes. The results showed that, except for the EG accessions, the diversity of wild *P. armeniaca* (DZG accessions) (nucleotide diversity and haplotype diversity: 0.0013, 0.444, respectively) was higher than that of the CAG (0.0006, 0.239) and NCG (0.0002, 0.400) accessions.

**Table 2:** Sample information and summary of haplotype/genotype distribution, genetic diversity for each population.

Population	cpDNA				ITS			
	Haplotype composition	N	*Hd*	π	Genotype composition	N	*Hd*	π
DZGhcmd	H1(6), H10(1), H11(1), H12(1)	9	0.583	0.0016	T2(2), T9(1), T14(1), T17(2), T19(1), T20(1), T21(1), T22(1)	10	0.956	0.0082
DZGhcy	H1(9)	9	0.000	0.0000	T3(2), T8(1), T9(3), T23(1),	7	0.810	0.0067
DZGhcm	H1(7), H8(1), H9(1)	9	0.417	0.0002	T2(1), T3(4), T8(1), T9(1), T18(1)	8	0.786	0.0000
DZGyn	H1(6), H7(1), H15(1), H16(1), H17(1)	10	0.667	0.0011	T1(1), T3(2), T8(2), T9(1), T14(2), T30(1)	9	0.917	0.0087
DZGglb	H1(6), H5(1), H6(1), H7(2)	10	0.644	0.0012	T2(1), T3(3), T8(2), T13(1), T14(1), T15(1)	9	0.889	0.0043
DZGgld	H1(6), H3(1)	7	0.286	0.0010	T3(4), T8(1), T16(1), T17(1)	7	0.714	0.0027
DZGxyt	H1(8), H2(1)	9	0.222	0.0001	T3(4), T8(2), T9(2), T12(1), T25(1)	10	0.822	0.0032
DZGxya	H1(1), H4(1), H13(1)	3	1.000	0.0039	T2(2), T24(1)	3	0.667	0.0988
DZGxyz	H1(7), H3(1), H14(1)	9	0.417	0.0031	T2(2), T3(2), T26(1), T27(1),T28(1), T29(2)	9	0.917	0.0057
Within population		75	0.444	0.0013		72	0.878	0.0055
CAG	H1(21), H2(1), H3(1), H4(1)	24	0.239	0.0006	T1(5), T2(11), T3(8), T4(1), T5(1), T6(1), T7(1), T8(1), T9(1), T10(1), T11(2), T12(1)	34	0.832	0.0053
NCG	H1(4), H2(1)	5	0.400	0.0002	T1(1), T3(5), T9(2), T48(1), T49(1), T50(2)	12	0.818	0.0047
EG	H1(1), H18(1), H19(1)	3	1.000	0.0045	T2(1), T3(4), T42(1)	6	0.600	0.0053
Within population		32	0.343	0.0012		52	0.833	0.0052
Among population		107	0.548	0.0019		124	0.865	0.0054
NAG	H28(1), H29(2)	3	0.667	0.0023	T2(1), T3(6), T8(1), T9(1), T12(1), T25(1), T44(1), T45(1), T46(1), T47(1)	15	0.857	0.0104
LX	H27(1)	1	0.000	0.0000	T3(4), T9(1)	5	0.400	0.0014
ZX	H33(1)	1	0.000	0.0000	T2(2), T55(1), T56(2), T57(2)	7	0.857	0.0229
ECG	H20(1)	1	0.000	0.0000	T11(1), T31(1), T32(1), T33(1), T34(1), T35(1), T36(1), T37(1), T38(1), T39(1), T40(1), T41(1)	12	1.000	0.017
ZHX	H32(1)	1	0.000	0.0000	T52(1), T53(1), T54(1)	3	1.000	0.0047
LMX	H23(1), H24(1), H25(1), H26(1)	4	1.000	0.0051	T3(3), T43(1)	4	0.500	0.0170
FGX	H21(2), H22(1)	3	0.667	0.0003				
T	H30(1), H31(1)	2	1.000	0.0005	T51(1)	1	0.000	0.0000

N, sample size; *Hd,* haplotype/genotype diversity; *π,* nucleotide diversity.

**Figure 2:**
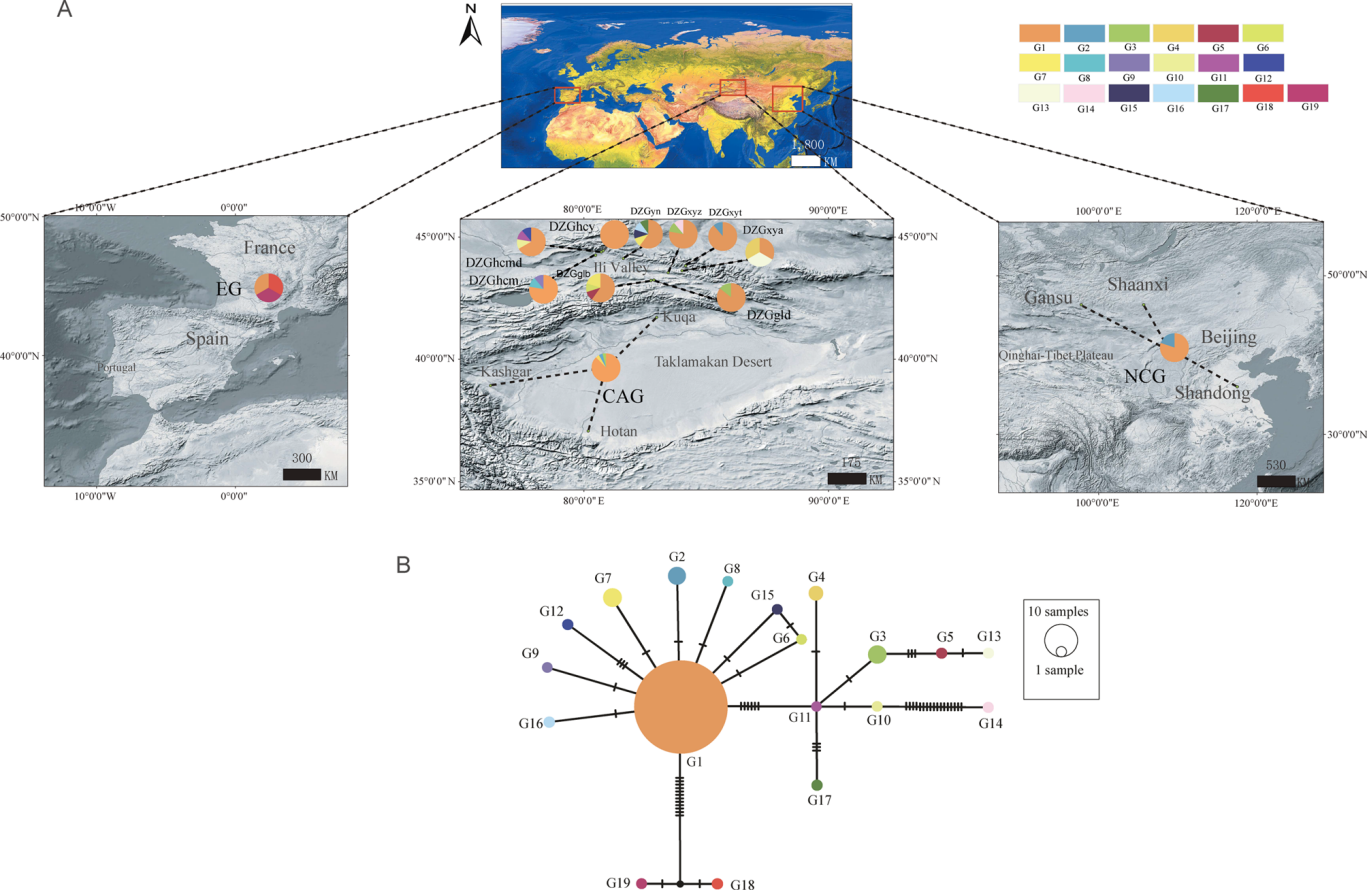
Geographic location and haplogroup distribution patterns of the 12 populations of *P. armeniaca* based on cpDNA dataset. (**A**) Geographic distribution of haplotyoes of *P. armeniaca*; Pie chart size corresponds to the sample size of each population. (**B**) The haplotype network generated from the haplotyoes of *P. armeniaca.* The haplotype network generated from the 19 haplotypes of *P. armeniaca*. The small black circles shown an intermediate haplotype not detected in this study. All maps (Source: http://ditu.ps123.net/world/2363.html) were generated using ArcGIS ver. 10.V.10.2.2 (ESRI, CA, USA) and then improved in Adobe Illustrator CC (Adobe Systems Inc., CA, USA).

In the ITS dataset, a total of 57 ITS genotypes were discovered among 171 individuals from 19 populations of *P. armeniaca* and its related species (Table 2, Figure S1), and the alignment length was 545 bp. The variable sites among the 57 genotypes are shown in Table S5. *P. armeniaca* harbored haplotypes T1–T30, T42 and T48–T50; *P. sibirica* harbored haplotypes T2–T3, T8–T9, T12, T25 and T44–T47; *P. mandshurica* harbored haplotypes T3 and T9; *P. dasycarpa* harbored haplotypes T2 and T55–T57; *P. mume* harbored haplotypes T11 and T31–T41; *P. zhengheensis* harbored haplotypes T52–T54; *P. limeixing* harbored haplotypes T3 and T43; and *P. davidiana* harbored haplotypes T51. Except for the EG accessions, the diversity of wild *P. armeniaca* (DZG accessions) (nucleotide diversity and haplotype diversity: 0.0055 and 0.878, respectively) was higher than that of the CAG (0.0053, 0.832) and NCG (0.0047 and 0.818) accessions (Fig. 2).

**Figure 3:**
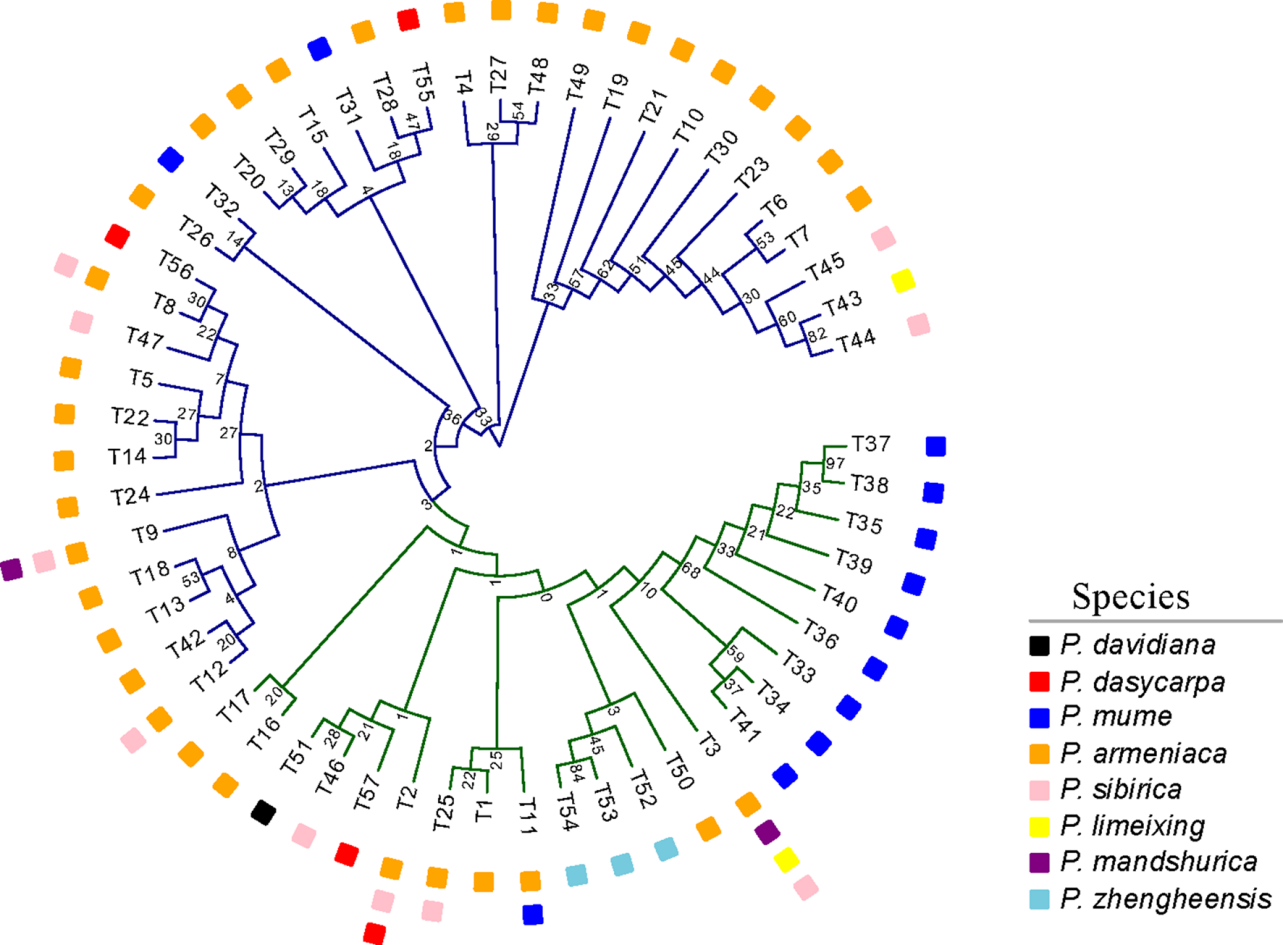
The Neighbor-joning consensus tree (NJ) of *Prunus* spp. based on genotypes of ITS sequences. The numbers on branches indicate the boostraps values.

A phylogenetic tree of all 57 genotypes was constructed to better understand their relationships (Fig. 3). The phylogenetic tree roughly divided the collected accessions into two groups. One group included the *P. armeniaca* (blue branch); the second group was composed of related species (green branch), including *P. sibirica*, *P. mandshurica*, *P. dasycarpa, P. mume, P. zhengheensis* and *P. limeixing.* T3 was widely distributed in most populations. The genetic backgrounds of the related species had the same genotypes (T2, T3, T11, T25, T8, T9, and T12) as *P. armeniaca*, indicating that they are associated with *P. armeniaca* through continuous and extensive gene flow.

Based on the concatenated cpDNA sequences (*trn*L*-trn*F and *ycf*1), 19 haplotypes (G1-G19) were identified among 107 individuals from 12 populations of *P. armeniaca* (Fig. 2). Variable sites among the 19 haplotypes are shown in Table S6. The *Hd* and π values detected at the cpDNA sequence level in *P. armeniaca* were 0.548 and 1.9 × 10^–3^, respectively. The geographic distribution of the 19 haplotypes is shown in Fig. 2A, illustrating that G1 haplotypes were distributed in all populations. The cpDNA haplotype network (Fig. 2B) showed that 7 haplotypes were differentiated from haplotype G1 by a one-step mutation with G1 at the center. Three haplotypes were differentiated from G11 by a one-step mutation. The overall network map showed a "star-like" distribution pattern. Based on the ITS sequences, 34 haplotypes (P1-P34) were identified among 124 individuals from 12 populations of *P. armeniaca* (Figure S2). The *Hd* and π values detected at the ITS sequence level in *P. armeniaca* were 0.865 and 5.4 × 10^–3^, respectively.

### Population structure and genealogical geography

In *P. armeniaca*, the gene diversity among populations (cpDNA: *H*_T_ = 0.499; ITS: *H*_T_ = 0.876) was higher than that within populations (cpDNA: *H*_S_ = 0.490; ITS: *H*_S_ = 0.794) (Table 3). A permutation test showed that *N*_ST_ was significantly higher than *G*_ST_ (cpDNA: *N*_ST_ = 0.227 > *G*_ST_ = 0.020; ITS: *N*_ST_ = 0.126 > *G*_ST_ = 0.094; *P* < 0.05), indicating that *P. armeniaca* has significant geographical structure (Table 3).

**Table 3:** Estimates of average gene diversity within populations (*H*_*S*_), total gene diversity (*H*_*T*_), interpopulation differentiation (*G*_*ST*_) and number of substitution types (*N*_*ST*_) for cpDNA haplotypes and ITS genotypes of *P. armeniaca*.

Populations	cpDNA				ITS			
	*H*_*S*_	*H*_*T*_	*G*_*ST*_	*N*_*ST*_	*H*_*S*_	*H*_*T*_	*G*_*ST*_	*N*_*ST*_
*P. armeniaca* (cultivated & wild)	0.490	0.499	0.020	0.227*	0.794	0.876	0.094	0.126*

Ns, not significant; *, P < 0.05.

AMOVA revealed significant genetic differentiation among all populations of *P. armeniaca* (cpDNA: *F*_*ST*_ = 0.1628, *P* < 0.001; ITS: *F*_*ST*_ = 0.0297, *P* < 0.001), with most of the genetic diversity occurring within the populations and relatively little occurring among them (Table 4).

**Table 4:** Analyses of molecular variance (AMOVA) of cpDNA haplotypes and ITS genotypes for populations of *P. armeniaca*.

Source of variation	cpDNA					ITS				
	*df*	*SS*	*VC*	*PV* (%)	*F*_*ST*_	*df*	*SS*	*VC*	*PV* (%)	*F*_*ST*_
Among populations	11	31.699	0.209	16.28		11	23.777	0.068	4.38	
Within populations	95	102.086	1.075	83.72		112	166.844	1.490	95.62	
Total	106	133.785	1.284		0.1628*	123	190.621	1.558		0.0297*

*df*, degrees of freedom; *SS*, sum of squares; *VC*, variance components; *PV*, percentage of variation; *F*_*ST*_, genetic diffrentiation; *P < 0.001.

### Demographic history and estimation of divergence times

**Figure 4:**
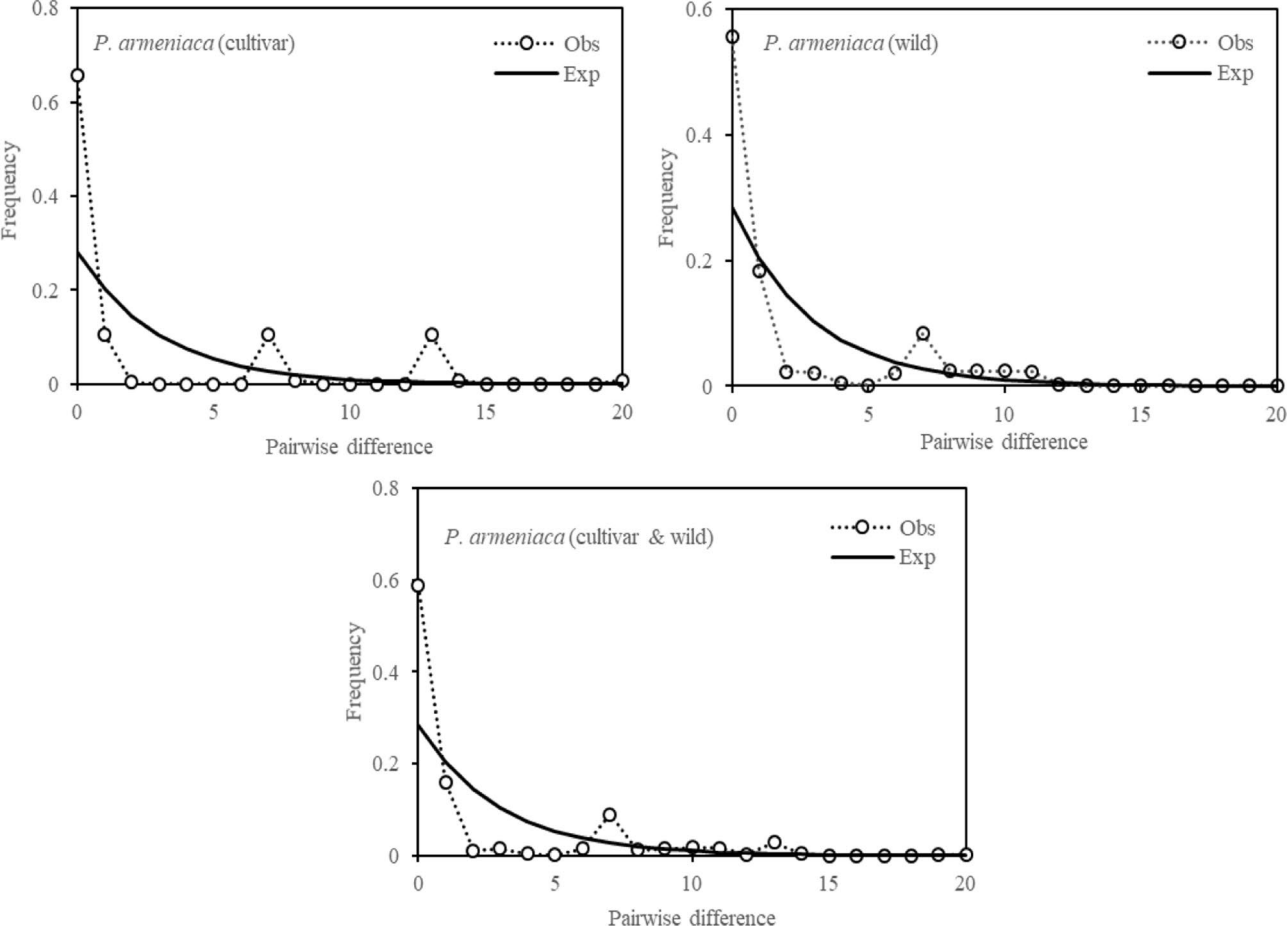
Mismatch distribution analysis of *P. armeniaca* based on overall gene pool of cpDNA dataset.

The mismatch distribution analysis based on the cpDNA and ITS dataset analysis, in which multimodal data were drawn from the cultivated populations or all populations, revealed a demographic equilibrium (Fig. 4, Figure S3). Both the neutrality tests based on Tajima’s *D* (cpDNA: − 2.272, *P* < 0.05; ITS: − 0.966, *P* < 0.05) and Fu’s *F*_*S*_ (cpDNA: − 5.8253, *P* < 0.05; ITS: -− 2.223, *P* < 0.05) and the mismatch distribution analysis (Fig. 4) based on the cpDNA and ITS datasets suggested recent range or demographic expansion in wild populations of *P. armeniaca*. In addition, neither the SSDs (cpDNA: 0.037, *P* > 0.05; ITS: 0.002, *P* > 0.05) nor the HRI (cpDNA: 0.179, *P* > 0.05; ITS: 0.017, *P* > 0.05) showed no significant positive values (Table 5), indicating no deviation of the observed mismatch distribution from that obtained via model simulation under sudden demographic expansion. Thus, we concluded that the demographic expansion of the wild populations of *P. armeniaca* occurred 16.53 kyr ago.

**Table 5:** Neutrality tests (Tajima’s *D* and Fu’s *F*_*S*_) and mismatch distribution analysis for the *P. armeniaca* based on the cpDNA and ITS dataset.

Populations		Tajima’s *D-*test		Fu’s *F*_*S*_*-*test		Mismatch distribution			
		*D*	*P*	*F*_*S*_	*P*	SSD	*P*	HRI	*P*
cpDNA	Overall	− 2.417	0.000*	− 6.350	0.025*	0.232	0.150	0.218	0.600
	*P. arminiaca* (cultivated)	− 1.933	0.003*	1.289	0.772	0.038	0.250	0.356	0.500
	*P. arminiaca* (wild)	− 2.272	0.001*	− 5.825	0.021*	0.037	0.070	0.179	0.880
ITS	Overall	− 1.193	0.106	− 8.587	0.010*	0.002	0.660	0.023	0.700
	*P. arminiaca* (cultivated)	− 1.420	0.155	− 4.951	0.024*	0.015	0.020	0.063	0.310
	*P. arminiaca* (wild)	− 0.966	0.041*	− 12.223	0.000*	0.002	0.810	0.017	0.880

*P < 0.05.

**Figure 5:**
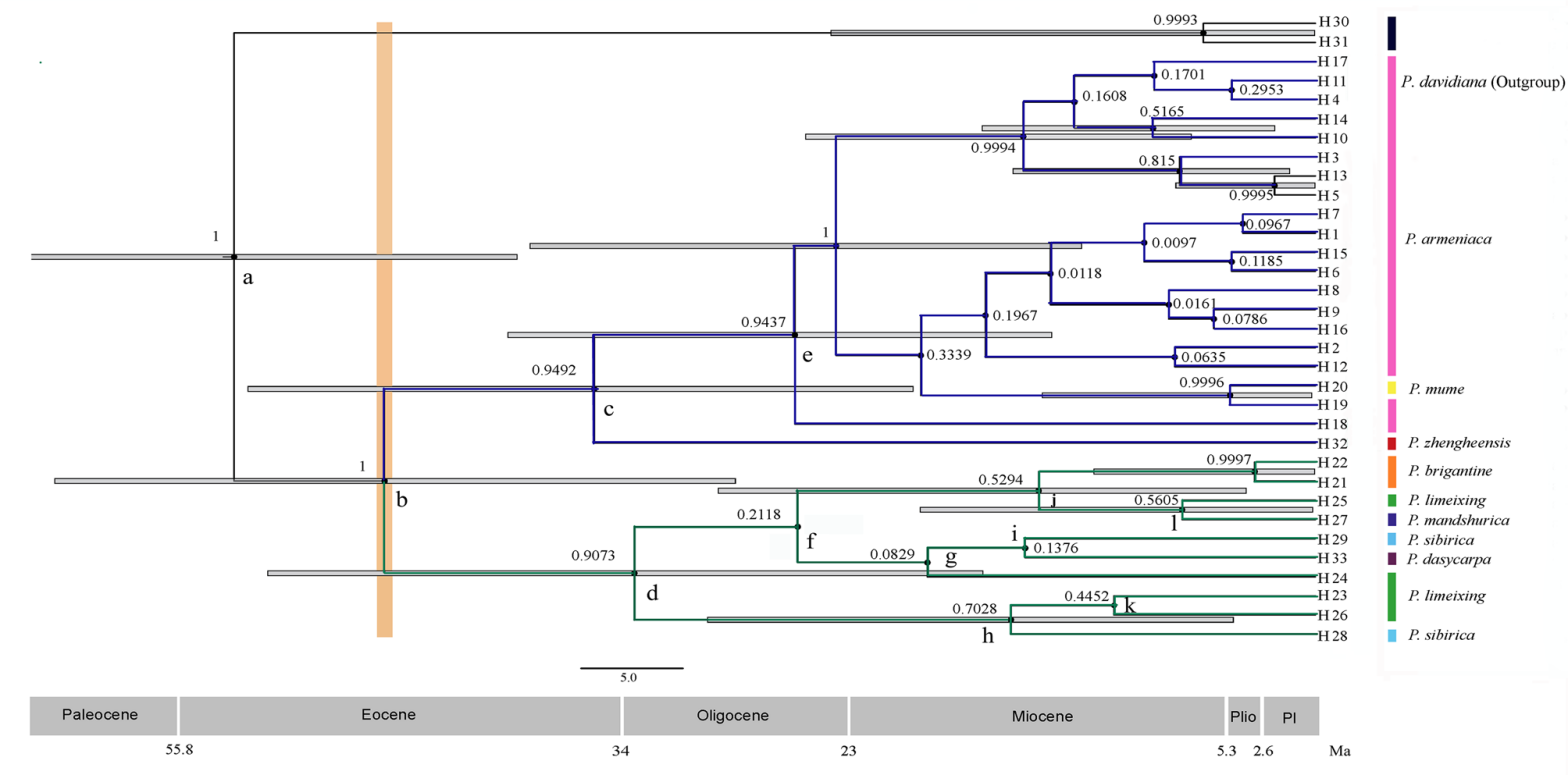
Best-derived chronogram of *Prunus* spp. based on chloroplast DNA. Gray bars represent 95% highest posterior density intervals. The numbers on the branches are posterior probabilities (PP > 0.9). Plio, Pliocene; Pl, Pleistocene; numbers above the branches indicate Bayesian posterior probabilities.

The cpDNA dataset was employed to estimate when the onset of divergence between *P. armeniaca* and its related species occrured (Fig. 5, Table S7). Thirty-three haplotypes were divided into two groups: those of *P. armeniaca* (blue) and those of related species (green) (Fig. 5). The divergence time estimation revealed that the differentiation of *P. armeniaca* from its related species occurred during the middle Eocene, approximately 45.68 Ma (95% highest posterior density (HPD) = 28.47–61.87). The onset of intraspecific divergence in *P. armeniaca* was estimated to have occurred 25.55 (95% HPD = 12.93–39.63) Ma.

## Discussion

Parental genetic markers are often combined with single-parent organelle markers for population genetics studies. Li et al.^32^ used cpDNA *trn*L-*trn*F, *rpl*16 and nrDNA ITS sequences to infer the evolutionary history of *S. sinomontana*. Yang et al.^52^ used cpDNA *psb*A-*trn*H, *trn*L-*trn*F, *ycf*1, and *mat*K sequences to access the demographical history and genetic diversity of a Deciduous Oak (*Quercus liaotungensis*) in Northern China. Zhang et al.^53^ used cpDNA markers to successfully determine the genetic diversity, genetic structure, and demographic history of 7 *Michelia yunnanensis* populations. Many scholars^54–57^ believed that the diversity of wild apricot is richest in the Ili River Valley, with low levels of genetic differentiation and genetic variation mainly occurring within populations. Hu et al.^56^ used simple sequence repeat markers to analyze the diversity of 212 apricot germplasms from 14 populations in the Ili River Valley. Among the populations, that from the Tuergen township in Xinyuan County had the highest genetic diversity, and the genetic distance between populations was significantly correlated with geographical distance. The self-incompatibility, wide distribution, and long-distance transmission of pollen through insects and strong winds of apricot are the main factors affecting its genetic structure^56^. Based on cpDNA and ITS data, we concluded that the haplotype/genotype diversity of wild apricot populations distributed in Ili River Valley was relatively high (Table 2), with that of the DZGhcmd and DZGyn populations being the highest. The results of AMOVA (Table 4) showed that the genetic diversity in *P. armeniaca* mainly occurs within populations (cpDNA: 83.72%; ITS: 97.03%), but there were also significant differences among populations (cpDNA: 16.28%; ITS: 2.97%), which was consistent with previous results based on simple sequence repeat markers^56^. The relatively high genetic diversity also confirmed the Tianshan Mountains as the origin center of cultivated apricot^56^. The limited informative mutation sites among the ITS genotypes led to very little resolution for the construction of genotype relationships (Figure S2), suggesting rapid intraspecific differentiation in the recently derived species *P. armeniaca*, similar to the results found in *S. sinomontana*^32^.

The genetic backgrounds of the related species had the same genotypes (T2, T3, T11, T25, T8, T9, and T12) as *P. armeniaca*, indicating that they are associated with *P. armeniaca* through continuous and extensive gene flow^57^. Liu et al.^58^ concluded that *P. sibirica* was divided into two groups based on microsatellite markers, one of which may have undergone gene exchange with *P. armeniaca*, further verifying our results. In addition, the authors found an extensively mixed genetic background in the germplasm of cultivated apricots in China.

This study indicated that the cultivated and wild populations of *P. armeniaca* had the same ancestral haplotype, G1. The haplotypes of the CAG, EG and NCG populations were mixed with the haplotypes/genotypes of the large wild populations (*P. armeniaca*). According to coalescent theory^59^, chloroplast haplotype G1, which was widely distributed and located in the center of the chloroplast network (Fig. 2), should be considered the oldest haplotype. The Kashgar, Hotan and Aksu oasis areas around the Tarim Basin in the southern part of the Xinjiang Uygur Autonomous Region of China are the main apricot-producing areas and contain the greatest abundance of apricot cultivars. There is only one mountain between southern Xinjiang and the Ili Valley, and there are several corridors between the northern and southern Tianshan Mountains. Therefore, the apricots cultivated in Xinjiang, southern Tianshan Mountains (CAG), most likely evolved from the spread of wild apricots in the Ili River Valley. Liu et al.^58^ argued that apricots have experienced at least three domestication events, giving rise to apricots in Europe (the United States and continental Europe), southern Central Asia (Turkmenistan, Afghanistan, and India) and China, with both ancient gene flow and recent gene mixing occurring. Central Asia harbors the highest diversity of wild apricots, with genetically differentiated populations that may have resulted from population isolation in glacial refugia^58^. In this study, the nucleotide diversity and haplotype/genotype diversity of wild apricots (DZG accessions) were higher than those of CAG and NCG accessions. These findings are reasonable from a historical perspective, as there was extensive cultural contact along the Silk Road from 207 BCE to 220 CE^60^. Therefore, historical and commercial influences may have contributed to the development of this unique species of cultivated apricot.

The theory and method of phylogeography can reveal the historical dynamics of species or populations, such as expansion, differentiation, isolation, migration and extinction^29^. It is of great significance for us to understand the origin of species and the evolution of geographical patterns, and to better protect existing biodiversity. Phylogeographic studies have shown that ancient haplotypes and high genetic diversity can be used to identify refuges^1,61^. Populations in refugia usually display more genetic diversity and exclusive haplotypes than migratory populations^14^. Many scholars^17,19,24^ have suggested that the complex geographic history of Northwest China may have provided refuges for species during glacial periods. By combining two markers, we showed that all the wild populations of apricots distributed in the Ili River Valley contained ancestral haplotypes/genotypes and had high genetic diversity (Table 2). These populations were located in areas considered glacial refugia for *P. armeniaca*, which appears to be a relic of Quaternary glaciation. The region provides a suitable climate for the biological community and protects the genetic diversity of *P. armeniaca*. Climatic changes during Pleistocene glacial-interglacial cycles had a dramatic effect on species distribution ranges, causing migration and/or extinction of populations, followed by periods of isolation, divergence and subsequent expansion^14^. Both neutrality tests and mismatch distribution analysis based on the cpDNA and ITS datasets suggested recent range or demographic expansion of wild populations of *P. armeniaca*. We estimated that the recent demographic expansion of the wild populations of *P. armeniaca* occurred 16.53 kyr ago, that is, at the end of the LGM^49^.

The selected taxon-sampling and fossil calibration strategies will influence the age estimation^62–63^. Due to the lack of fossil evidence for *P. armeniaca*, we used the peach-apricot divergence time as the calibration point. In this study, we tried to use a cpDNA dataset to estimate divergence time, and the effect was acceptable. However, compared with the median ages estimated by Chin et al.^50^ (mean age of 31.1 Ma), the divergence time estimates in this study should be interpreted with caution because the limited coverage and low number of calibration points may lead to an overly high divergence time estimate for *P. sibirica* (mean age of 33.43 Ma).

## Conclusion

Based on cpDNA and ITS data, the haplotype/genotype diversity of wild apricot populations distributed in the Ili River Valley was relatively high, and the haplotype/genotype diversity of DZGhcmd and DZGyn populations was greater than that of other populations. *P. armeniaca* exhibits genealogical structure. Affected by the Quaternary glaciation of the Pleistocene, the Ili River Valley in Northwest China served as a glacial refugium for *P. armeniaca*, providing the species with a suitable climate and preserving its genetic diversity. During the interglacial period, the species underwent a recent expansion in the face of favorable climatic and environmental conditions. Apricots originated during the middle Eocene, and the cultivated apricot in Xinjiang originated from apricots in the Ili River Valley in Northwest China.

### Research involving plants

The experimental research and field studies on plants in this work comply with the IUCN Policy Statement on Research Involving Species at Risk of Extinction and the Convention on the Trade in Endangered Species of Wild Fauna and Flora.

## Supplementary Information

Supplementary Information 1.

Supplementary Information 2.

Supplementary Information 3.

Supplementary Information 4.

Supplementary Information 5.

Supplementary Information 6.

Supplementary Information 7.

Supplementary Information 8.

Supplementary Information 9.
