# Supplementary material for: Phylogeography of Prunus armeniaca L. revealed by chloroplast DNA and nuclear ribosomal sequences
Source: Sci Rep. 2021 Jul 1;11:13623. doi: 10.1038/s41598-021-93050-w (PMC8249649; doi:10.1038/s41598-021-93050-w)
Supplement: Supplementary file 1 — Supplementary Information 1. [file 41598_2021_93050_MOESM1_ESM.docx]

Phylogeography of *Prunus armeniaca* L. by Chloroplast DNA and Nuclear Ribosomal Sequences

Wen-Wen Li^1^, Li-Qiang Liu^1^, Qiu-Ping Zhang^2^, Wei-Quan Zhou^1^, Guo-Quan Fan^3^, Kang Liao^1*🖂^

^1^College of Horticulture and Forestry, Xinjiang Agricultural University, Urumqi, Xinjiang, China. ^2^Xiongyue National Germplasm Resources Garden of the Liaoning Institute of Pomology, Xiongyue, Shenyang, China. ^3^Luntai National Fruit Germplasm Resources Garden of Xinjiang Academy of Agricultural Sciences, Luntai, Xinjiang, China. **^🖂^**[email](mailto:email): [liaokang01@163.com](mailto:liaokang01@163.com).
