# Supplementary material for: Phylogeography of Prunus armeniaca L. revealed by chloroplast DNA and nuclear ribosomal sequences
Source: Sci Rep. 2021 Jul 1;11:13623. doi: 10.1038/s41598-021-93050-w (PMC8249649; doi:10.1038/s41598-021-93050-w)
Supplement: Supplementary file 2 — Supplementary Information 2. [file 41598_2021_93050_MOESM2_ESM.docx]

**Figure**


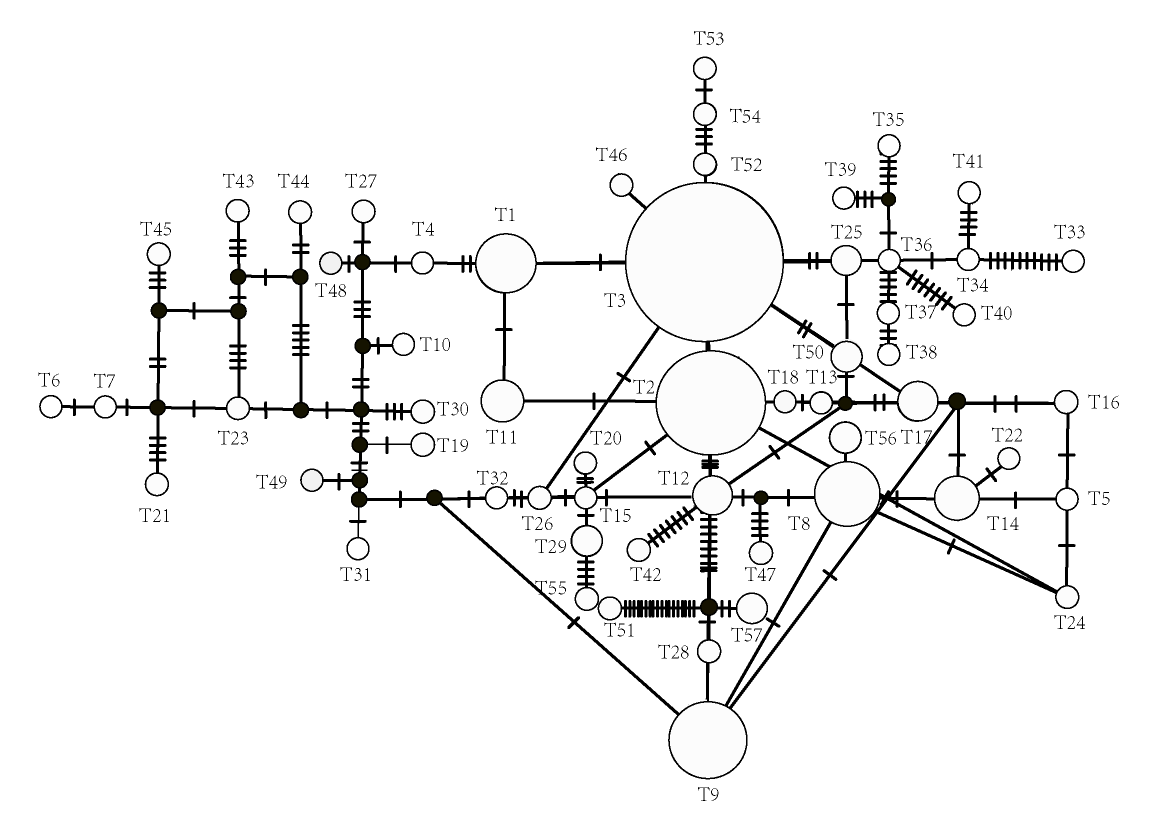


Figure S1 The haplotype network generated from the haplotypes of *Prunus armeniaca* and related species based on ITS dataset. The small black circles shown an intermediate haplotype not detected in this study.


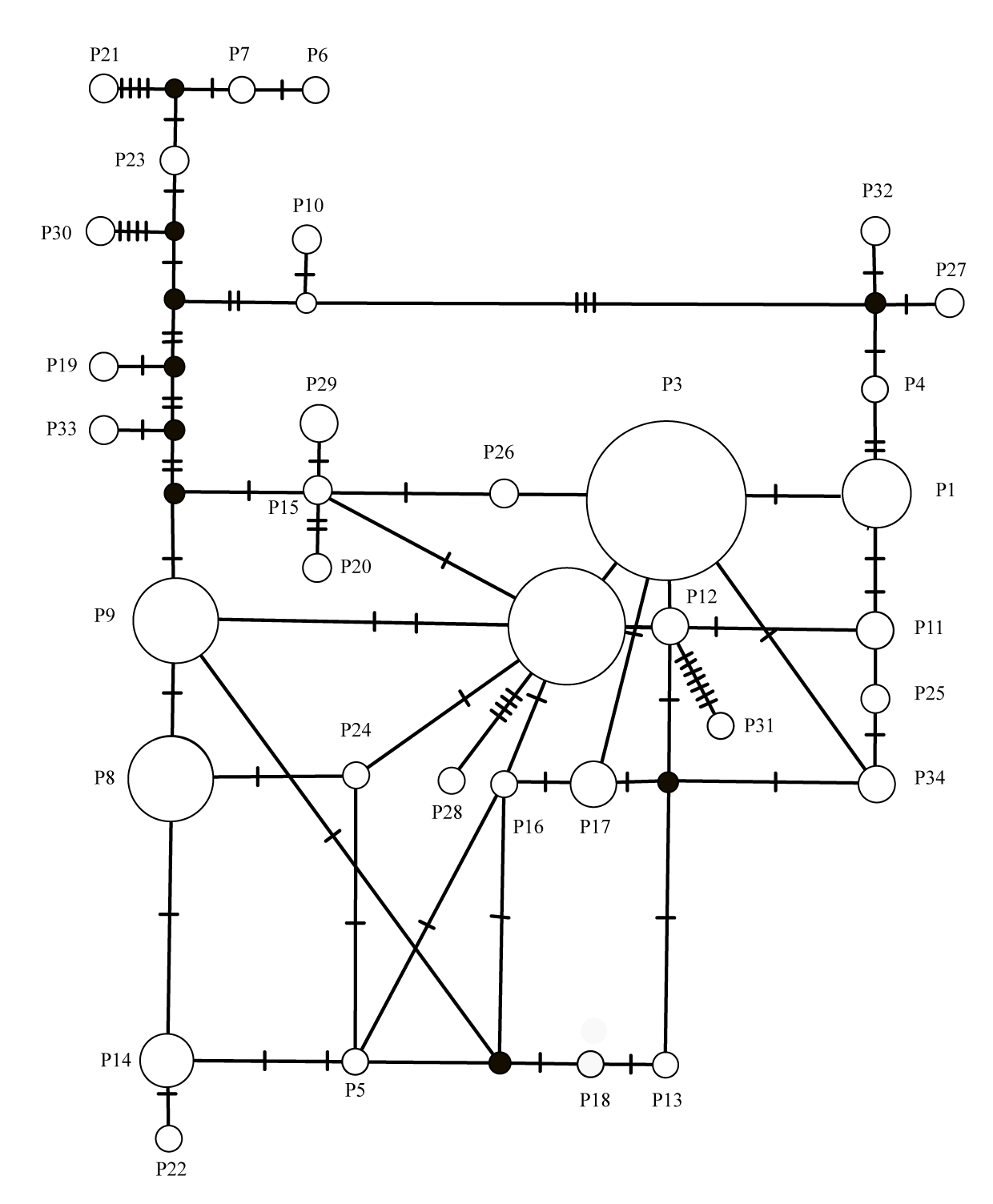


Figure S2 The haplotype network generated from the haplotypes of *P. armeniaca* based on ITS dataset. The small black circles shown an intermediate haplotype not detected in this study.


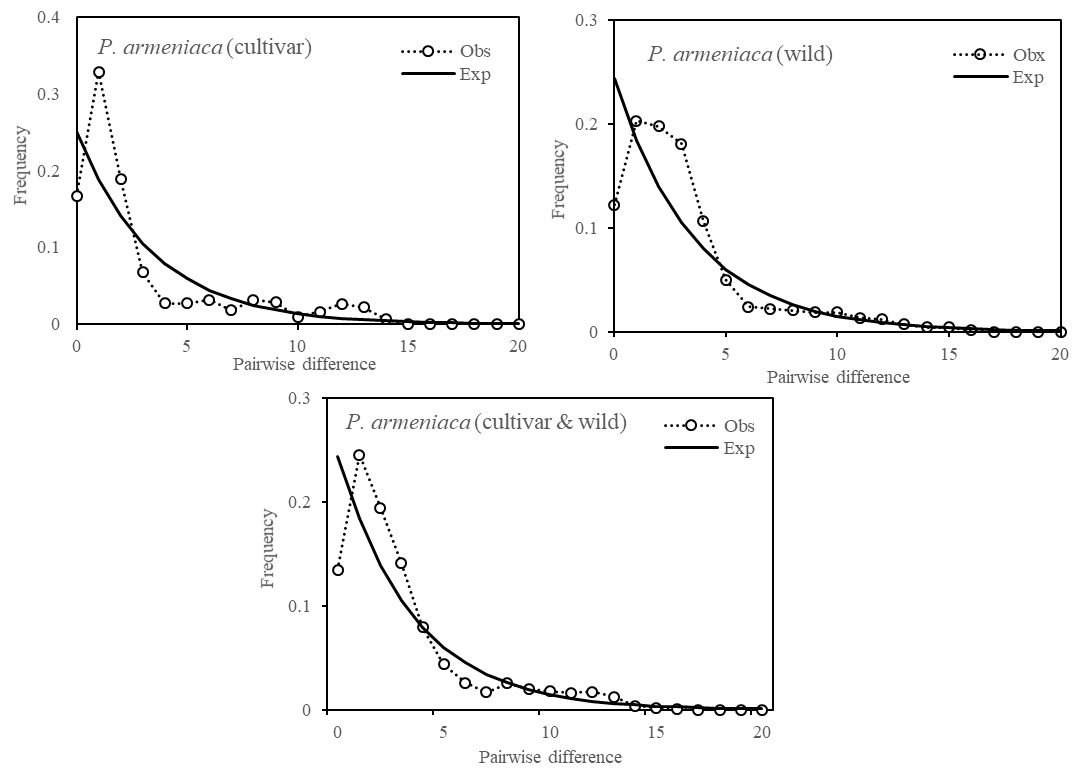


Figure S3 Mismatch distribution analysis of *P. armeniaca* based on ITS data.
