# Supplementary material for: Phylogeography of Prunus armeniaca L. revealed by chloroplast DNA and nuclear ribosomal sequences
Source: Sci Rep. 2021 Jul 1;11:13623. doi: 10.1038/s41598-021-93050-w (PMC8249649; doi:10.1038/s41598-021-93050-w)
Supplement: Supplementary file 3 — Supplementary Information 3. [file 41598_2021_93050_MOESM3_ESM.docx]

Table S1 The gene number of NCBI database used for analysis in this study.

| **Species** | **No. of ITS** | **Species** | **No. of ITS** |
| --- | --- | --- | --- |
| *P. mume* | AF185616.1 | *P. percica* | JF978121.1 |
| *P. mume* | EF211073.1 | *P. sibirica* | AF318739.1 |
| *P. mume* | EF211074.1 | *P. sibirica* | JF978137.1 |
| *P. mume* | EF211075.1 | *P. sibirica* | JF978138.1 |
| *P. mume* | EF523482.1 | *P. sibirica* | JF978139.1 |
| *P. mume* | EF523483.1 | *P. sibirica* | JF978140.1 |
| *P. mume* | EF523485.1 | *P. sibirica* | MG735463.1 |
| *P. mume* | EF523486.1 | *P. sibirica* | MG772981.1 |
| *P. mume* | EF523487.1 | *P. sibirica* | MH711531.1 |
| *P. mandshurica* | JF978108.1 | *P. sibirica* | JQ034171.1 |
| *P. mandshurica* | EF211082.1 | *P. sibirica* | FJ980390.1 |
| *P. zhengheensis* | JF978141.1 | *P. armeniaca* | EF211085_1 |
| *P. zhengheensis* | JF978142.1 | *P. armeniaca* | KX890454_1 |
| *P. dasycarpa* | KX890449.1 | *P. armeniaca* | KX890455_1 |
| *P. dasycarpa* | KX890449.1 | *P. armeniaca* | KX890457_1 |
| *P. dasycarpa* | KX890450.1 | *P. armeniaca* | KX890458_1 |
| *P. dasycarpa* | KX890451.1 | *P. armeniaca* | MG735461_1 |
| *P. dasycarpa* | KX890452.1 | *P. armeniaca* | MG735477_1 |
| *P. dasycarpa* | KX890452.1 | *P. armeniaca* | MG735479_1 |
