# Supplementary material for: Phylogeography of Prunus armeniaca L. revealed by chloroplast DNA and nuclear ribosomal sequences
Source: Sci Rep. 2021 Jul 1;11:13623. doi: 10.1038/s41598-021-93050-w (PMC8249649; doi:10.1038/s41598-021-93050-w)
Supplement: Supplementary file 4 — Supplementary Information 4. [file 41598_2021_93050_MOESM4_ESM.docx]

Table S2 Voucher information for *Prunus* spp. used in this study.

| **Population** | **Voucher localities** | **Voucher data** | **Collector’s Name** | **Voucher number** |
| --- | --- | --- | --- | --- |
| *P. armeniaca* |  |  |  |  |
| DZGhcmd | Huocheng, Xinjiang | 26 April 2019 | Wenwen Li, Liqiang Liu, Kang Liao | DZGhcmd |
| DZGhcy | Huocheng, Xinjiang | 26April 2019 | Wenwen Li, Liqiang Liu, Kang Liao | DZGhcy |
| DZGhcm | Huocheng, Xinjiang | 26 April 2019 | Wenwen Li, Liqiang Liu, Kang Liao | DZGhcm |
| DZGyn | Yining, Xinjiang | 25 April 2019 | Wenwen Li, Liqiang Liu, Kang Liao | DZGyn |
| DZGglb | Gongliu, Xinjiang | 24 April 2019 | Wenwen Li, Liqiang Liu, Kang Liao | DZGglb |
| DZGgld | Gongliu, Xinjiang | 24 April 2019 | Wenwen Li, Liqiang Liu, Kang Liao | DZGgld |
| DZGxyt | Xinyuan, Xinjiang | 23 April 2019 | Wenwen Li, Liqiang Liu, Kang Liao | DZGxyt |
| DZGxya | Xinyuan, Xinjiang | 23 April 2019 | Wenwen Li, Liqiang Liu, Kang Liao | DZGxya |
| DZGxyz | Xinyuan, Xinjiang | 23 April 2019 | Wenwen Li, Liqiang Liu, Kang Liao | DZGxyz |
| CAG | Luntai, Xinjiang | 27 April 2019 | Wenwen Li, Liqiang Liu, Kang Liao | CAG |
| NCG | Luntai, Xinjiang | 27 April 2019 | Wenwen Li, Liqiang Liu, Kang Liao | NCG |
| EG | Xiongyue, Liaoning | 27 May 2019 | Qiuping Zhang | EG |
| *P. sibirica* |  |  |  |  |
| NAG | Xiongyue, Liaoning | 27 May 2019 | Qiuping Zhang | NAG |
| *P. mandshurica* |  |  |  |  |
| LX | Xiongyue, Liaoning | 27 May 2019 | Qiuping Zhang | LX |
| *P. dasycarpa* |  |  |  |  |
| ZX | Luntai, Xinjiang | 27 April 2019 | Wenwen Li, Liqiang Liu, Kang Liao | ZX |
| *P. mume* |  |  |  |  |
| ECG | Xiongyue, Liaoning | 27 May 2019 | Qiuping Zhang | ECG |
| *P. zhengheensis* |  |  |  |  |
| ZHX | Xiongyue, Liaoning | 27 May 2019 | Qiuping Zhang | ZHX |
| *P. limeixing* |  |  |  |  |
| LMX | Xiongyue, Liaoning | 27 May 2019 | Qiuping Zhang | LMX |
| *P. brigantine* |  |  |  |  |
| FGX | Xiongyue, Liaoning | 27 May 2019 | Qiuping Zhang | FGX |
| *P. davidiana* |  |  |  |  |
| T | Luntai, Xinjiang | 27 April 2019 | Wenwen Li, Liqiang Liu, Kang Liao | T |
