# Supplementary material for: Phylogeography of Prunus armeniaca L. revealed by chloroplast DNA and nuclear ribosomal sequences
Source: Sci Rep. 2021 Jul 1;11:13623. doi: 10.1038/s41598-021-93050-w (PMC8249649; doi:10.1038/s41598-021-93050-w)
Supplement: Supplementary file 5 — Supplementary Information 5. [file 41598_2021_93050_MOESM5_ESM.docx]

Table S3 Primer pairs of cpDNA and ITS sequences used in this study.

| **Sequence fragment** | | | **Primers sequence (5'-3')** |
| --- | --- | --- | --- |
| cpDNA | *trn*L*-trn*F | | F: GGGCGATCCTGAGCCAAAT |
|  |  |  | R: ATCCCGACCAGTCACGACA |
|  | *ycf*1 | | F:GCGCATCAGTGGAGTTATAGGAA |
|  |  |  | R:GCGCATTAGTTTCTTGGGTATTG |
|  | | ITS | F: TCCTCCGCTTATTGATATGC |
|  |  |  | R: GGAAGTAAAAGTCGTAACAAGG |

*F, the forward primer; R, the reverse primer.*
