# Supplementary material for: Phylogeography of Prunus armeniaca L. revealed by chloroplast DNA and nuclear ribosomal sequences
Source: Sci Rep. 2021 Jul 1;11:13623. doi: 10.1038/s41598-021-93050-w (PMC8249649; doi:10.1038/s41598-021-93050-w)
Supplement: Supplementary file 9 — Supplementary Information 9. [file 41598_2021_93050_MOESM9_ESM.docx]

Table S7 The differentiation time of *P. armeniaca* and related species based on cpDNA dataset.

| **Node label** | **Ma** |
| --- | --- |
| a | 53.0701 |
| b | 45.6823 |
| c | 35.3884 |
| d | 33.4257 |
| e | 25.5480 |
| f | 25.4041 |
| g | 19.0376 |
| h | 14.9338 |
| i | 14.2402 |
| j | 13.5802 |
| k | 9.8792 |
| l | 6.5375 |
